# Bushen-Yizhi formula ameliorates cognition deficits and attenuates oxidative stress-related neuronal apoptosis in scopolamine-induced senescence in mice

**DOI:** 10.3892/ijmm.2014.1801

**Published:** 2014-06-11

**Authors:** XUE-QIN HOU, DIAN-WEI WU, CHUN-XIA ZHANG, RONG YAN, CONG YANG, CUI-PING RONG, LEI ZHANG, XIANG CHANG, RU-YU SU, SHI-JIE ZHANG, WEN-QING HE, ZHAO QU, SHI LI, ZI-REN SU, YUN-BO CHEN, QI WANG, SHU-HUAN FANG

**Affiliations:** 1 Institute of Clinical Pharmacology, Guangzhou University of Chinese Medicine, Guangzhou, Guangdong 510405, P.R. China; 2 Shantou Hospital of Traditional Chinese Medicine, Guangzhou, Guangdong 515031, P.R. China; 3 School of Chinese Materia Medica, Guangzhou University of Chinese Medicine, Guangzhou, Guangdong 510006, P.R. China

**Keywords:** Alzheimer’s disease, scopolamine, Bushen-Yizhi formula, antioxidant, antiapoptosis, neuroprotection

## Abstract

Bushen-Yizhi formula (BSYZ), a traditional Chinese medicine formula consisting of six herbs has been reported to possess a neuroprotective effect. The present study aimed to investigate the effects of BSYZ on learning and memory abilities, as well as oxidative stress and neuronal apoptosis in the hippocampus of scopolamine (SCOP)-induced senescence in mice, in order to reveal whether BSYZ is a potential therapeutic agent for Alzheimer’s disease (AD). A high-performance liquid chromatography (HPLC) fingerprint was applied to provide a chemical profile of BSYZ. Extracts of BSYZ were orally administered to mice with SCOP-induced memory impairment for two weeks. The learning and memory abilities were determined by the Morris water maze test. The oxidant stress-related indices, such as activity of superoxide dismutase (SOD) and levels of glutathione (GSH) and malondialdehyde (MDA) were examined in hippocampus of SCOP-treated mice. The cell death ratio was assessed by TUNEL staining, while apoptotic-related proteins including Bcl-2 and Bax were determined by immunofluorescent staining and western blot analysis. Caspase-3 was determined by western blot analysis. Consequently, a chromatographic condition, which was conducted at 35°C with a flow rate of 0.8 ml/min on the Gemini C18 column with mobile phase of acetonitrile and water-phosphoric acid (100:0.1, v/v), was established to yield common fingerprint chromatography under 203 nm with a similarity index of 0.986 within 10 batches of BSYZ samples. BSYZ at a dose of 2.92 g/kg significantly improved the cognitive ability, restored the abnormal activity of SOD and increased the levels of MDA and GSH induced by SCOP. Moreover, the neural apoptosis in the hippocampus of SCOP-treated mice was reversed by BSYZ by regulating the expression of Bcl-2, Bax and caspase-3. The results demonstrated that BSYZ had neuroprotective effects in SCOP-induced senescence in mice by ameliorating oxidative stress and neuronal apoptosis in the brain, supporting its potential in AD treatment.

## Introduction

Alzheimer’s disease (AD) is a progressive neurodegenerative disease accompanied by neuronal loss in the brain and cognitive impairments. The exact pathogenesis is still unknown although several theories have been revealed and accepted. Cholinergic system dysfunction was one of the most important pathogenesis of AD (1–3). Acetylcholinesterase inhibitor, a target drug of the cholinergic system, has been used to treat mild to moderate AD in clinical trials in recent years (4,5). Scopolamine (SCOP), an anticholinergic agent, has been reported to induce some features that are similar to AD, such as affective disorder and memory impairment, and was widely used to induce the cognitive impairment model for investigation of AD (6–8). Studies have shown that it induced oxidative stress in SCOP-treated animals (9). Oxidative stress, another important pathogenesis of AD (10), was associated with neuronal loss or apoptosis (11,12). Since the pathogenesis is complicated, the target of therapy is diverse. Multi-targeted drugs play an increasingly important role in the treatment of AD (13). Chinese medicine recipes, comprising multi-components, serve as a potential multi-targeted drug. Certain compound recipes (14–16) exert beneficial effects on cognitive ability in memory-impaired animal models and are crucial in the clinical therapy of AD patients. In the traditional Chinese medicine theory, deficiency of kidney was considered to be the root cause of AD. Thus, the therapy of reinforcing kidney was widely used in the clinic by using ‘kidney-reinforcing’ herbs or prescription (17) which were reported to possess anti-AD effects. Moreover, different herbs had different effects on brain functions, such as antioxidant, anti-apoptotic and anti-acetylcholinesterase activities (18,19). The Bushen-Yizhi formula (BSYZ) is a traditional Chinese medicine compound recipe consisting of common *Cnidium* fruit (CCF), tree peony bark (TPB), ginseng root (GR), Radix Polygoni Multiflori Preparata (RPMP), barbary wolfberry fruit (BWF) and Fructus Ligustri Lucidi (FLL). In traditional Chinese medicine theory, BSYZ had an effect of ‘kidney-reinforcing’ and ‘brain nourishing’ based on the single effect of those six herbs as well as the effect of compatibility of traditional Chinese medicine, which was considered to play a more important role in treatment of disease. Previous studies have shown that the medicine-containing serum of BSYZ exerted effects of enhancing choline acetyltransferase (ChAT) activity and neurotransmitter release in a cell model of Aβ25–35-induced AD (20,21). However, additional evidence is required to reveal the potential therapeutic effects of BSYZ in AD. In this study, we investigated the effects of BSYZ extraction on improving cognitive disorder in SCOP-induced senescence in mice by Morris water maze test, a common method for assessing learning and memory abilities of animals. Additionally, the effects of BSYZ on oxidative stress-related apoptosis were investigated to illuminate the underlying mechanisms.

## Materials and methods

### Materials

Ginsenoside Rb1, Ginsenoside Rg1, Osthole, Imperatorin, Paeoniflorin, Paeonolum, Oleanic acid and 2,3,5,4′-tetrahydroxystilbene-2-O-β-D-glucoside were purchased from the National Institutes for Food and Drug Control (Beijing, China). Acetonitrile [high performance liquid chromatography (HPLC) grade] was bought from Honeywell International Inc. (Burdick & Jackson, Muskegon, MI, USA). SCOP hydrobromide injection (Guangzhou Baiyun mountain Mingxing Pharmaceutical Co., Ltd., Guangzhou, China) was purchased from Guangzhou Pharmaceuticals Corporation (Guangzhou, China). Acricept (Henan Joyline & Joysun Pharmaceutical Stock Co., Ltd., Zhengzhou, China) was dissolved in 0.9% physiological saline. Kits used for determination of superoxide dismutase (SOD), malondialdehyde (MDA) and glutathione (GSH) were purchased from the Nanjing Jiancheng Bioengineering Institute (Nanjing, China). Primary antibodies (Bcl-2, caspase-3 and β-actin) were obtained from Cell Signaling Technology, Inc. (Beverly, MA, USA). Anti-Bax antibody was purchased from Santa Cruz Biotechnology, Inc. (Santa Cruz, CA, USA). Secondary antibodies (horseradish peroxidase-conjugated anti-rabbit IgG and anti-mouse IgG) were purchased from Cell Signaling Technology, Inc. Other reagents were of AR grade.

### Preparation of sample solution

BSYZ consisted of six medicinal plants (Table I). All the raw herbs were purchased from the Guangxi Yifang Chinese Herbal Medicine Department and identified by Professor Jiannan Chen, pharmacognosist of the School of Chinese Materia Medica, Guangzhou University of Chinese Medicine. All of these accorded with the standard described in the 2010 edition of China Pharmacopoeia. The contents of BSYZ or dried powder of single herb were weighed and subjected to an ultrasonic extraction with 60 ml of 70% methanol for 30 min. The extract solution was then filtered through a 0.45 μm filter membrane prior to analysis.

### HPLC analysis

The HPLC equipment was Dionex Summit HPLC system, equipped with a PDA-100 detector, a P680 pump, an ASI-100 automatic sampler, and a STH585 thermostatic column compartment. The chromatographic separation was carried out at 35°C with a flow rate of 0.8 ml/min on a Gemini-C18 110A (150×2.00 mm, 5 μm). The mobile phase was A (acetonitrile) and B (water-phosphoric acid, 100:0.1, v/v), and 10 μl capacity per injection was used. The elution program was optimized and conducted as follows: 0–23 min, linear gradient 5–19% A; 23–33 min, linear gradient 19–22% A; 33–48 min, linear gradient 22–32% A; 48–60 min, linear gradient 32–75% A; 60–61 min, linear gradient 75–80% A; 61–66 min, linear gradient 80% A; 66–78 min, linear gradient 80–5% A; and 78–80 min, linear gradient 5% A. Monitoring was performed at 203 nm with PDA detector. Data analysis was performed by a similarity evaluation system for chromatographic fingerprint of traditional Chinese medicine (Version 2004A, The Pharmacopoeia Commission of PRC, Beijing, China), which was recommended by the State Food and Drug Administration (SFDA) of China. The software was usually used to evaluate the similarities of different chromatograms and calculate the correlative coefficient of different patterns.

### Animals and drug administration

Male Kunming mice (8-month-old, weighing 50–60 g) were purchased from the Experimental Animal Center of Sun Yat-Sen University (Guangzhou, China). Mice were maintained on standard laboratory conditions with food and water *ad libitum* for the duration of the study. The animal experiments were approved by the Animal Ethics Committee of Guangzhou University of Chinese Medicine. Mice were randomly divided into six groups (n=9): the vehicle control group (0.9% NaCl treatment), SCOP group (SCOP 2 mg/kg), Aricept group (SCOP + Aricept 3 mg/kg), low dose BSYZ group (SCOP + BSYZ 1.46 g/kg), medium dose BSYZ group (SCOP + BSYZ 2.92 g/kg) and high dose BSYZ group (SCOP + BSYZ 5.84 g/kg). Mice were orally administered saline, Aricept or BSYZ, once per day for two weeks. In the vehicle control and SCOP groups, mice were treated similarly with corresponding volumes of saline. The mice, with the exception of the vehicle control group, were intraperitoneally administered SCOP 30 min prior to the Morris water maze test.

### Morris water maze test

The Morris water maze test was similar to the method of Morris (22), with minor modifications (23). The equipment (Guangzhou Feidi Biology Technology Co., Ltd., Guangzhou, China) consisted of a black circular pool (120 cm in diameter and 40 cm in height), filled to a depth of 30 cm with water (22–26°C) and a non-toxic water-soluble black colored dye. The pool was divided into four equal quadrants and a black escape platform (8 cm in diameter, 1 cm below the water surface) was placed in the center of one of the pool quadrants. The learning and memory ability of mice was detected by the Morris water maze test in a dark room. Mice were given a place navigation test for five consecutive days. On each training day, there were four sequential training trials for each mouse from four different entry positions equally spaced around the perimeter of the pool. A trial began by placing the animal in the water facing the wall of the pool at one starting point and the escape latency was recorded at the end. If it failed to find the platform within 60 sec, the mouse was guided to the platform by the experimenter and allowed to stay there for 20 sec and its escape latency was recorded as 60 sec. After four trials, the mouse was dried and returned to its cage at the end. On the sixth day, the probe test was performed in the absence of the platform with a cut-off time of 60 sec. The number of crossing through the original position of the platform and the time spent in the target quadrant were measured.

### Biochemical analysis (assay of SOD activity, MDA and GSH level)

After the Morris water maze test, six mice from each group were anesthetized and decapitated. Brains were removed carefully and dissected into hippocampus and cortex on an ice-cold plate. Tissues were rapidly stored at −80°C until use. Parts of samples were used for biochemical analysis and western blot analysis.

For the biochemical analysis, the hippocampus was weighed and homogenized with ice-cold saline in a glass homogenizer to make 10% (weight/volume) tissue homogenate. Homogenate was centrifuged at 3,000 × g for 10 min at 4°C and the supernatant was used to assay SOD activity, MDA and GSH contents by using the commercial kits according to the manufacturer’s instructions. The absorbance was read at 550, 532 and 420 nm, respectively, using Universal Microplate Spectrophotometer (Bio-Rad, Hercules, CA, USA). The levels of SOD activity, MDA and GSH contents were expressed as U/mg protein, nmol/mg protein and μg/mg protein, respectively.

### Preparation of sections

Three mice from each group were anesthetized and decapitated. Brains were removed carefully and quickly fixed in 4% paraformaldehyde in 0.1 M phosphate-buffered saline (PBS, pH 7.4) for 24 h, dehydrated with a graded series of ethanol, embedded in paraffin blocks and sliced at 4 μm thickness.

### TUNEL staining

TUNEL staining was performed using the In Situ Cell Death Detection kit (Roche Diagnostics GmbH, Mannheim, Germany), according to the manufacturer’s instructions. Briefly, the sections were heated at 60°C for 1 h, washed in xylene and rehydrated through a graded series of ethanol and double-distilled water. After treating sections with 0.1 M citrate buffer (pH 6.0) by microwave oven for 1 min and cooling them to room temperature, the sections were washed in PBS and incubated with 50 μl TUNEL reaction mixture for 1 h at 37°C in the dark. Further incubation with 50 μl converter-POD was performed at 37°C for 30 min. The sections were then rinsed with PBS and stained with DAB substrate for 10 min at room temperature. Images were captured and analyzed at a magnification of ×200 by using a light microscope and LEICA QWin plus (Leica Microsystems, Wetzlar, Germany). Average TUNEL-positive cells of each animal were obtained from three adjacent sections.

### Immunofluorescent staining of Bcl-2 and Bax proteins

Sections were dewaxed and rehydrated by conventional methods. After quenching endogenous peroxidase with 3% hydrogen peroxide for 10 min and blocking with normal goat serum for 10 min at 37°C, sections were incubated with rabbit anti-Bax antibody (1:200) and mouse anti-Bcl-2 antibody (1:200) (both from Santa Cruz Biotechnology, Inc.) at 4°C overnight. After washing in PBS, the sections were incubated with FITC-conjugated anti-rabbit IgG (1:500) and Cy3 conjugated anti-mouse IgG (1:200) (both from Beijing Cowin Biotech Co., Ltd., Beijing, China) for 1 h at room temperature in the dark. Images were captured at a magnification of ×200 for analysis. The mean fluorescence intensity (MFI) was measured, and expression levels of Bax and Bcl-2 were calculated as change of the percentage in MFI compared to the vehicle control mice.

### Western blot analysis

For western blot analysis assay, the hippocampus was homogenized and lysed in ice-cold RIPA buffer (containing 1:100 PMSF, 1:100 inhibitor proteases and phosphatases cocktail) for 15 min. The lysate was centrifuged at 12,000 × g for 15 min at 4°C and the supernatant was removed to a new 1.5 ml centrifuge tube. The protein concentrations were detected according to the manufacturer’s instructions of the BCA protein assay kit (Nanjing Biobox Biotech. Co., Ltd., Nanjing, China). Samples (40 μg of protein) were subjected to SDS-PAGE analysis in 12% gel. The separated protein was then transferred to PVDF membranes. The membranes were blocked with 5% non-fat milk dissolved in Tris-buffered saline-Tween-20 (TBST) for 1 h at room temperature and subsequently incubated with rabbit anti-Bcl-2 (1:2,000, Cell Signaling Technology, Inc.), rabbit anti-Bax (1:2,000, Santa Cruz Biotechnology, Inc.), rabbit anti-caspase-3 (1:2,000) and mouse anti-β-actin (1:5,000) (both from Cell Signaling Technology, Inc.) overnight at 4°C. The membranes were subsequently washed three times in TBST for 10 min each time and then incubated with horseradish peroxidase-conjugated anti-rabbit or anti-mouse IgG antibody (diluted at 1:5,000) for 1 h at room temperature. After washing the membranes in TBST three times, immunopositive bands were visualized using a super-enhanced chemiluminescense western blot analysis-detection reagent (ECL; Applygen Technologies Inc, Beijing, China). The optical density (OD) of bands on X-ray film was determined. β-actin was used as internal control. Results were expressed as the percentage of OD values by using the Image J2x software system.

### Statistical analysis

Data were shown as the mean ± SE and analyzed using the Statistical Package for Social Science (SPSS) 17.0 software. In the Morris water maze test, escape latency was analyzed using repeated measures analysis of variance (ANOVA). When the Mauchly’s test was significant, the differences between pairs of means were assessed by the multivariate analysis together with the least significant difference (LSD) post-hoc test. Other data obtained from the Morris water maze, and biochemical, TUNEL and western blot analyses were analyzed using one-way ANOVA. Values of P<0.05 were considered to be statistically significant.

## Results

### HPLC analysis of the main components in BSYZ

**Figure 1:**
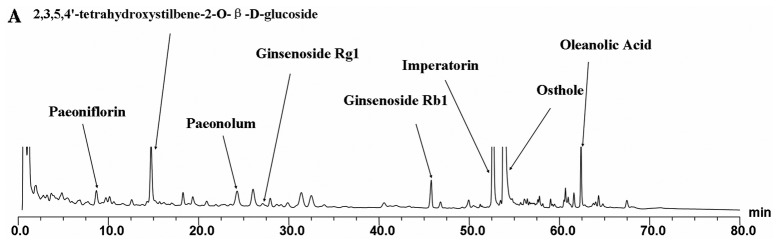

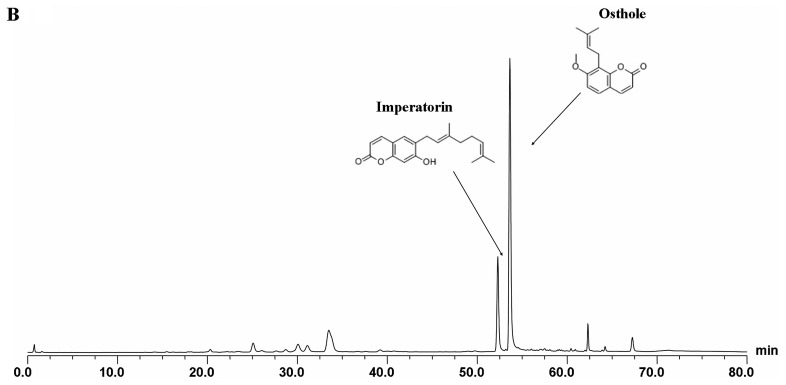

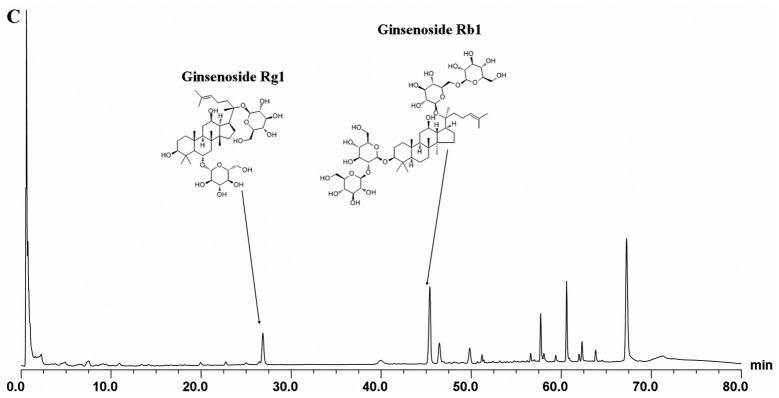

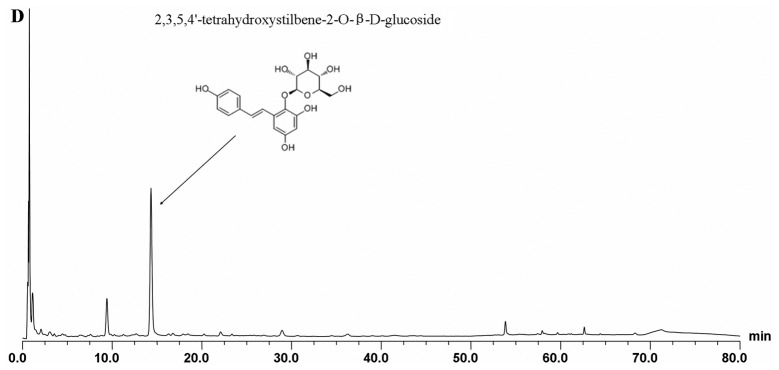

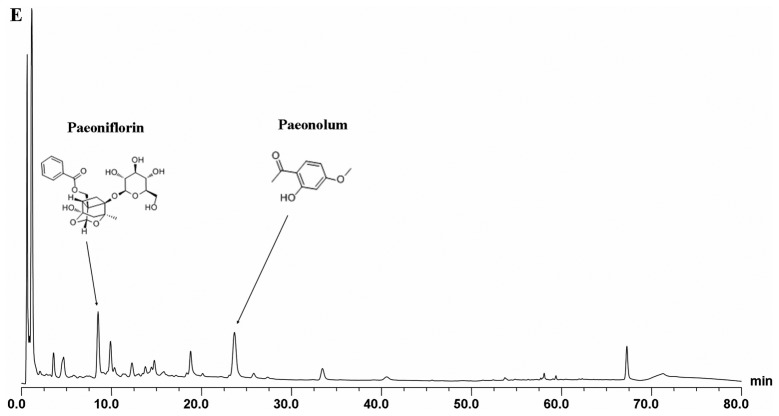

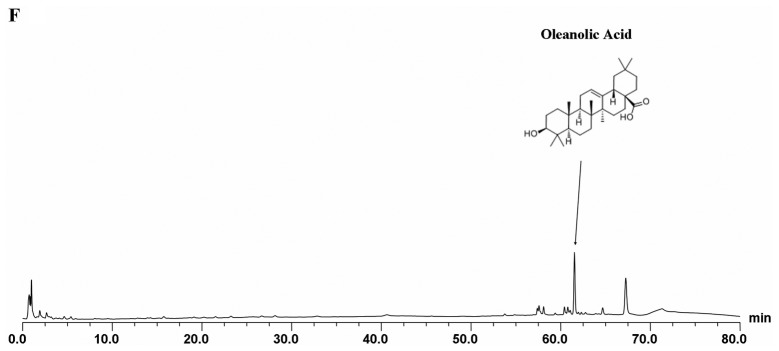

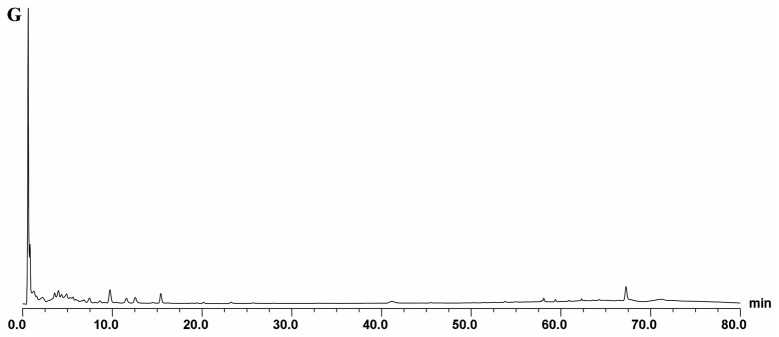
High-performance liquid chromatography (HPLC) pattern of Bushen-Yizhi formula (BSYZ) and single herbal extracts. (A) Structures of the constituents identified from BSYZ, (B) She Chuang Zi (*Cnidium monnier*i L. Cuss., fruit), (C) Ren Shen (*Panax ginseng* C. A. Mey., rhizome), (D) Zhi He Shou Wu (Preparata of *Polygonum multiflorum* Thuna., radix). (E) Mu Dan Pi (*Paeonia suffruticosa* Andr., cortex), (F) Nv Zhen Zi (*Ligustrum lucidum* Ait., fruit), (G) Gou Qi (*Lycium barbarum* L., fruit).

**Figure 2:**
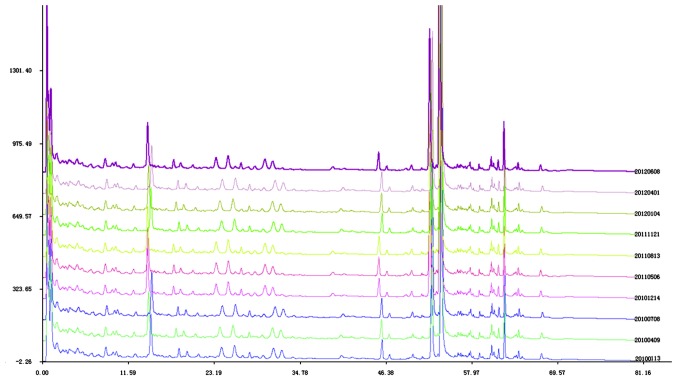
The chromatogram of 10 batches of Bushen-Yizhi formula (BSYZ) for similarity evaluation.

The proposed HPLC analytical method was applied to acquiring the fingerprint of different batches of BSYZ samples. HPLC fingerprint of BSYZ is shown in Figs. 1 and 2. The relative retention time (RRA) and relative peak area (RPA) of all common peaks, whose relative standard deviation (RSD) values were ≤3.7%, were obtained with reference to this substance. The results indicated the good stability and reproducibility of the fingerprint analysis by HPLC. The similarity indices of 10 batches of BSYZ samples were calculated using a similarity evaluation system. The results demonstrated that the samples showed good correlation and shared a similar chromatographic pattern with the similarity indices at >0.986. By comparing the retention times and UV spectra of the reference standards, eight compounds (Paeoniflorin, 2,3,5,4′-tetrahydroxystilbene-2-O-β-D-glucoside, Paeonolum, Ginsenoside Rg1, Ginsenoside Rb1, Imperatorin, Osthole, and Oleanic acid) in BSYZ were identified.

### Morris water maze test

**Figure 3:**
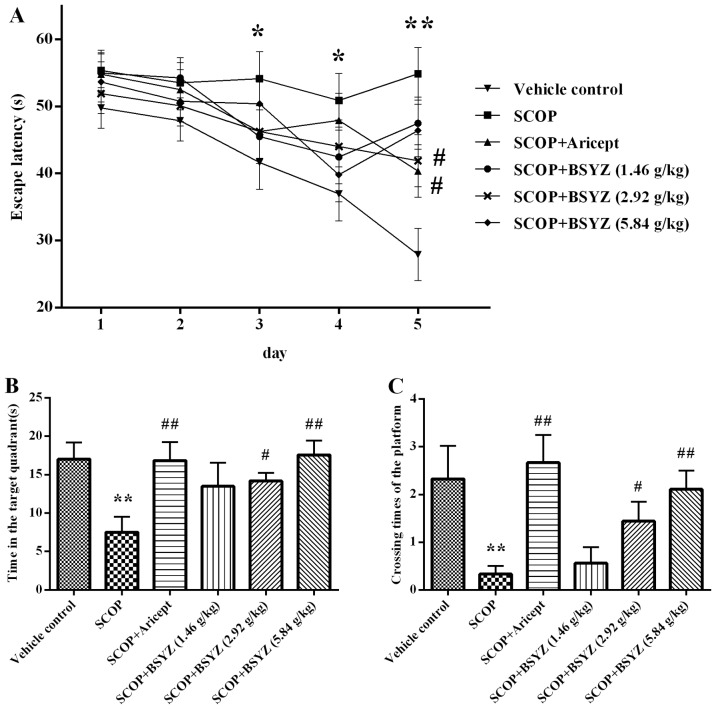

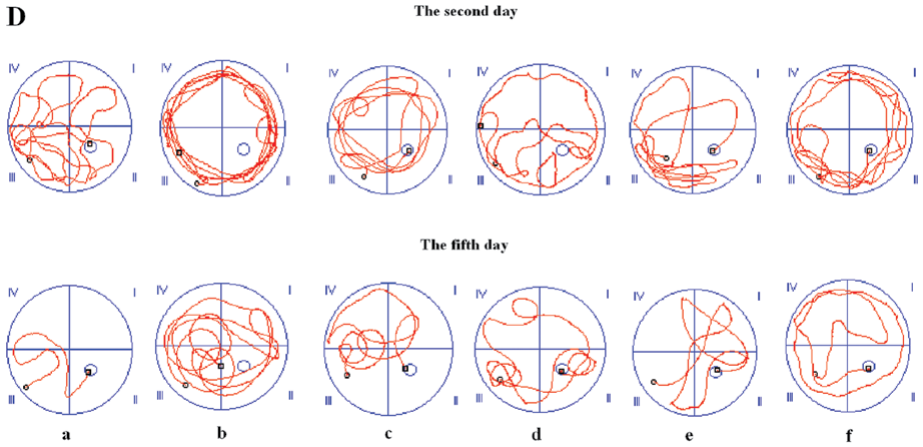
The Morris water maze test of mice. (A) Escape latencies in the water maze during five consecutive days test. (B) Time spent in the quadrant of platform placed within 60 sec. (C) Crossing times of the platform location. (D) Represent tracks of mice in the fourth trial on the second and fifth days; a, Vehicle control; b, SCOP; c, SCOP + Aricept; d, SCOP + Bushen-Yizhi formula (BSYZ) (1.46 g/kg); e, SCOP + BSYZ (2.92 g/kg); f, SCOP + BSYZ (5.84 g/kg) group. Data are shown as the mean ± SE. ^*^P<0.05 and ^**^P<0.01 versus the vehicle control mice. ^#^P<0.05 and ^##^P<0.01 versus the SCOP-treated mice.

The spatial learning and memory ability of mice was tested by the Morris water maze test. As shown in Fig. 3A, the escape latency declined progressively during the five training days. The SCOP-treated mice spent longer period of time in finding the platform than the vehicle control mice from the third to fifth days (P<0.05, P<0.05 and P<0.01, respectively). These results revealed that the SCOP-treated mice had significant cognitive impairment. Moreover, Aricept (3 mg/kg)- and BSYZ (2.92 g/kg)-treated mice significantly shortened the escape latency compared with the SCOP-treated mice on the fifth day (both P<0.05). In the spatial probe test, the time spent in the target quadrant and the crossing times of the platform location were showed as Fig. 3B and C. Compared with the vehicle control group, SCOP-treated mice spent less time (P<0.01) in the target quadrant and crossed to the platform fewer times (P<0.01). In the Aricept (3 mg/kg)- and BSYZ (2.92 and 5.84 g/kg)-treated mice, the test revealed a significant increase both in time spent in the quadrant of the platform placed and crossing counts compared with the SCOP-treated mice (P<0.01, P<0.05 and P<0.01, respectively). Fig. 3D showed the swim tracks of mice in the fourth trial of the second and fifth days in the place navigation test. Mice tended to swim in circles around the wall of the pool on the second day. The mice gradually changed this search strategy within five training days. On the fifth day, SCOP-treated mice took a longer period of time and complex swimming tracks were noted, while the vehicle control mice swam in the direction of the platform. Aricept- and BSYZ-treated mice performed similar tracks to the vehicle control mice.

### Effect of BSYZ on the SOD activity and MDA, GSH levels

The antioxidant effects of BSYZ in SCOP-treated mice are shown in Table II. SCOP treatment induced SOD activity decrease of 36.75% in the hippocampus. However, Aricept and BSYZ (2.92 g/kg) treatment resulted in a significant elevation of enzyme activity in SCOP-treated mice, by increases of 39.37 and 34.82%, respectively. The MDA level in the hippocampus of SCOP-treated mice induced an increase of 88% more than the vehicle control group (P<0.01). This increase was reversed by treatment with Aricept and BSYZ (2.92 g/kg), with a percentage of 36.17 and 44.68%, respectively. The GSH content significantly decreased in the hippocampus of SCOP-treated mice compared with the vehicle control mice (P<0.01). Aricept and BSYZ (2.92 and 5.84 g/kg) treatment induced increases of the GSH level in the hippocampus of ~1.54-, 1.84-, and 1.48-fold, respectively, compared with the SCOP-treated mice.

### Effect of BSYZ on neuronal apoptosis in the hippocampus

**Figure 4:**
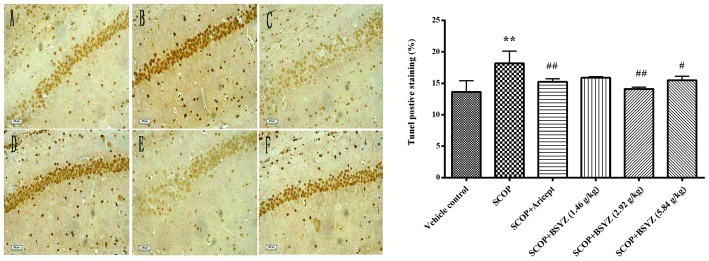
TUNEL staining in the hippocampus of scopolamine (SCOP)-treated mice. (A) Vehicle control; (B) SCOP; (C) SCOP + Aricept; (D) SCOP + Bushen-Yizhi formula (BSYZ) (1.46 g/kg); (E) SCOP + BSYZ (2.92 g/kg); (F) SCOP + BSYZ (5.84 g/kg); scale bar, 200 μm; original magnification, ×200. Ratio of TUNEL-positive neurons is shown as the mean ± SE (n=3). ^**^P<0.01 versus the vehicle control mice. ^#^P<0.05 and ^##^P<0.01 versus the SCOP-treated mice.

Representative images of the TUNEL staining in hippocampus are shown in Fig. 4. TUNEL-positive cells were stained a deep brown in the hippocampus. Cell counting of the neuronal apoptosis in the hippocampus of SCOP-treated mice (18.18±1.94%, P<0.01) was prominently more than in the vehicle control mice (13.82±1.78%). Aricept and BSYZ (2.92 and 5.84 g/kg) treatments markedly attenuated neuronal apoptosis in SCOP-treated mice (15.24±0.47%, P<0.01; 14.11±0.26%, P<0.01; and 15.48±0.64%, P<0.05, respectively). These results indicated that BSYZ attenuated SCOP-induced apoptotic cell death in the hippocampus.

### Effect of BSYZ on the expression of Bax and Bcl-2 proteins by immunofluorescent staining

**Figure 5:**
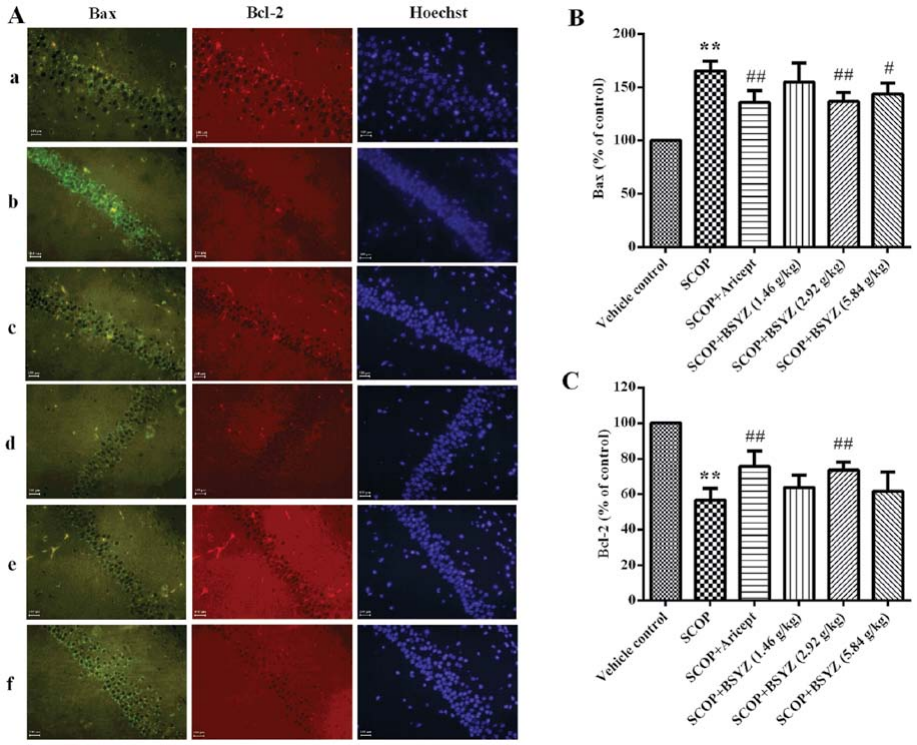
Immunofluorescent staining of Bax and Bcl-2 in the hippocampus of mice. (A) The expression of Bax and Bcl-2 in the hippocampus of SCOP-treated mice analyzed by immunofluorescent staining (scale bar, 100 μm; original magnification, ×400). Bax was labeled with FITC (green) and Bcl-2 was labeled with Cy3 (red). a, Vehicle control; b, SCOP; c, SCOP + Aricept; d, SCOP + Bushen-Yizhi formula (BSYZ) (1.46 g/kg); e, SCOP + BSYZ (2.92 g/kg); f, SCOP + BSYZ (5.84 g/kg). (B) Percentage in mean fluorescence intensity (MFI) of Bax to the vehicle control mice. (C) Percentage in MFI of Bcl-2 compared to the control mice. Data are shown as the mean ± SE (n=3). ^**^P<0.01 versus the vehicle control mice. ^#^P<0.05 and ^##^P<0.01 versus the SCOP-treated mice.

The expression of Bax and Bcl-2 in the hippocampus of SCOP-treated mice was analyzed by immunofluorescent staining (Fig. 5A). As shown in Fig. 5B and C, the MFI of Bax in the hippocampus of SCOP-treated mice was higher than the vehicle control group (165.40±9.20% of control, P<0.01), while MFI for Bcl-2 was lower (56.73±6.57% of control, P<0.01). Following treatment with BSYZ at a dose of 2.92 g/kg, MFI of Bax was markedly reduced (136.72±8.26% of control, P<0.01), while Bcl-2 was significantly elevated (73.59±4.49% of control, P<0.01). Treatment with Aricept showed a similar effect with BSYZ.

### Effect of BSYZ on the expression of Bax, Bcl-2 and caspase-3 proteins by western blot analysis

**Figure 6:**
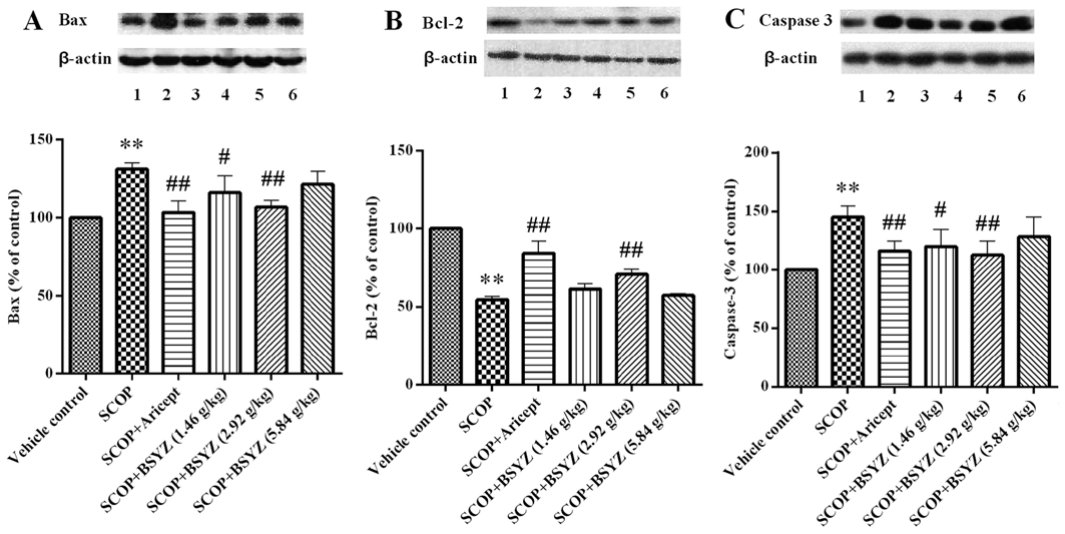
Effect of Bushen-Yizhi formula (BSYZ) on the expression of Bax, Bcl-2 and caspase-3 proteins. A, B and C show the expression of Bax, Bcl-2 and caspase-3 proteins, respectively; lanes 1, Vehicle control; 2, SCOP; 3, SCOP + Aricept; 4, SCOP + BSYZ (1.46 g/kg); 5, SCOP + BSYZ (2.92 g/kg); 6, SCOP + BSYZ (5.84 g/kg); Data are shown as the mean ± SE (n=3). ^**^P<0.01 versus the vehicle control mice. ^#^P<0.05 and ^##^P<0.01 versus the SCOP-treated mice.

The expression of Bax, Bcl-2 and caspase-3 proteins in the hippocampus of SCOP-treated mice was analyzed by western blot analysis. As shown in Fig. 6, the mean OD of Bcl-2 was lower in the SCOP-treated mice than the vehicle control group (P<0.01), while the mean optical densities of Bax and caspase-3 were higher than the vehicle control group (P<0.01). Following treatment with BSYZ at dose of 2.92 g/kg, the mean OD of Bcl-2 was significantly elevated (P<0.01), and the mean densities of Bax and caspase-3 were markedly reduced (P<0.01). The same effect was observed in the Aricept treatment group. In addition, BSYZ treatment at a dose of 1.46 g/kg showed a downregulated effect on the expression of Bax and caspase-3 proteins (P<0.05).

## Discussion

The gradual decline of learning and memory is a typical symptom of AD. Findings of previous studies have shown that a similar symptom was induced by SCOP (24), which is known as a cholinergic receptor antagonist. SCOP-treated animals have been widely used to estimate memory impairment and screening of potential cognition-enhancing agents. Oxidative stress and apoptosis changes, which are considered to be two important factors of pathogenesis of AD, occur in this model (6,25,26). In this study, we evaluated the effects of BSYZ on SCOP-induced memory impairments in mice by using Morris water maze test, biochemical assessments of oxidative stress indices and expression of apoptosis related proteins.

The Morris water maze test is widely used to evaluate the spatial learning and memory ability of animals (6,9,27). In this study, it was shown that SCOP treatment prolonged the escape latency, shortened the time spent in the target quadrant and reduced the crossing times of the platform location compared with the control group. This indicates that the animals had a cognitive dysfunction. Moderate to high dose of BSYZ treatment showed a reverse effect similar to Aricept (also known as Donepezil) which is an acetylcholinesterase inhibitor and was approved to be used to treat mild and moderate dementia patients by the US Food and Drug Administration (FDA) in 1996. It has been widely used in studies of AD (28–30) and has shown antioxidant and anti-apoptotic activities (31).

Concerning oxidative stress, there was an imbalance of antioxidant systems, such as a decrease of SOD activity and GSH level, and an increase in the MDA level. In this study, the assessments of oxidative stress indices showed that SCOP-treated mice possessed a decreased SOD activity and GSH levels as compared to the control mice, while MDA levels were elevated. However, Aricept and BSYZ treatment increased SOD activity and the GSH level while reducing the MDA level. These results indicate that BSYZ may have potent antioxidant activity by exerting a protective effect against oxidative damage induced by SCOP.

Apoptosis, another important pathogenesis of AD, has been reported to be associated with the mechanism of central cholinergic system dysfunction and oxidative stress. In the present study, we found that SCOP markedly increased neuronal apoptosis in the hippocampus by TUNEL staining. BSYZ and Aricept treatment improved this brain damage.

Numerous genes are involved in the regulation of the mitochondrial apoptotic pathway. The proto-oncogene Bcl-2 is an inhibitor of apoptosis protein which exerts anti-apoptotic effects. In our study, SCOP significantly reduced the expression of Bcl-2 protein in the hippocampus of mice, while BSYZ and Aricept both induced an upregulation.

Bax, a pro-apoptotic protein, exerts an opposite effect to Bcl-2. It was reported that a high expression of Bax protein promoted cell death. The ratio of Bcl-2 to Bax determines the susceptibility of cell apoptosis. Caspase-3, a key executioner of apoptosis in programmed cell death, was able to induce neuronal dysfunction (32). Studies have shown that an increase of Bcl-2 and a decrease of Bax prevented the release of cytochrome *c* in mitochondria, and therefore inhibit the cascade of apoptosis. Our results demonstrate that BSYZ arrested the upregulation of Bax in the hippocampus of SCOP-treated mice, which enhanced the modulation of apoptosis. Moreover, the results show that BSYZ reversed the elevation of caspase-3 activity in the hippocampus of SCOP-treated mice by western blot analysis.

Recently, multi-targeted therapy is employed in various diseases, particularly those associated with different pathogenesis. In traditional Chinese medicine, plant extracts from different herbs in a formula may contain different ingredients, which may play a different role in treating the same disease. Furthermore, it was reported that different ingredients potentiated each other’s effect (33). It was identified by HPLC that BSYZ consisted of eight major components, including Ginsenoside Rb1, Ginsenoside Rg1, Osthole, Imperatorin, Paeoniflorin, Paeonolum, Oleanic acid and 2,3,5,4′-tetrahydroxystilbene-2-O-β-D-glucoside, from its six herbs respectively. The pharmacological effects of the components were various, such as antioxidant, neuroprotective effect, anti-apoptotic and anti-inflammatory (34–37). Moreover, it has been reported that some of the components had beneficial effects in the improvement of learning and memory ability (38,39).

Findings of our study suggest that BSYZ exerted a neuroprotective effect in SCOP-treated mice. The mechanism involved was presumably the regulation of oxidative stress and the expression of mitochondrial-mediated apoptosis-related proteins. However, it is essential to perform a further study to elucidate the detailed mechanism.

In summary, SCOP induced learning and memory impairment of mice. Moreover, BSYZ effectively improved cognitive ability and restored the abnormal activity of SOD and levels of MDA and GSH, reversed neural apoptosis, downregulated the expression of Bax and caspase-3 and upregulated the expression of Bcl-2 in the hippocampus. These data suggest that BSYZ exerted enhancing cognitive function, which may result from the regulation of the antioxidative defense system and mitochondrial-mediated apoptosis mechanism. BSYZ is therefore a potential therapeutic agent for AD. However, future investigations should be conducted to demonstrate the effects of BSYZ on AD.

## Figures and Tables

**Table I:** Constituents of BSYZ.

Components	Ratio
She Chuang Zi (*Cnidium monnieri* L. Cuss., fruit)	3
Ren Shen (*Panax ginseng* C. A. Mey., rhizome)	3
Zhi He Shou Wu (Preparata of *Polygonum multiflorum* Thuna., radix)	2
Mu Dan Pi (*Paeonia suffruticosa* Andr., cortex)	2
Nv Zhen Zi (*Ligustrum lucidum* Ait., fruit)	2
Gou Qi (*Lycium barbarum* L., fruit)	2

BSYZ, Bushen-Yizhi formula.

**Table II:** Effects of BSYZ on SOD activity, and MDA and GSH content in the hippocampus of scopolamine-treated mice.

Group	SOD (U/mg protein)	MDA (nmol/mg protein)	GSH (μg/mg protein)
Vehicle control	45.22±1.61	0.25±0.11	5.68±1.14
SCOP	28.60±4.42^a^	0.47±0.14^a^	2.81±0.52^a^
SCOP + Aricept (3 mg/kg day)	39.89±7.02^b^	0.30±0.14^b^	4.34±0.96^b^
SCOP + BSYZ (1.46 g/kg day)	34.68±6.24	0.38±0.08	3.40±0.45
SCOP + BSYZ (2.92 g/kg day)	38.56±8.00^b^	0.26±0.04^c^	5.18±0.40^c^
SCOP + BSYZ (5.84 g/kg day)	30.50±3.02	0.35±0.07	4.17±0.76^b^

BSYZ, Bushen-Yizhi formula; SOD, superoxide dismutase; MDA, malondialdehyde; GSH, glutathione. Data are shown as the mean ± SE (n=4).

a P<0.01 versus the vehicle control mice,

b P<0.05 and

c P<0.01 versus the scopolamine-treated mice.
